# Seasonal parasitism and host specificity of *Trissolcus japonicus* in northern China

**DOI:** 10.1007/s10340-017-0863-y

**Published:** 2017-04-18

**Authors:** Jinping Zhang, Feng Zhang, Tara Gariepy, Peter Mason, Dave Gillespie, Elijah Talamas, Tim Haye

**Affiliations:** 1 MoA-CABI Joint Laboratory for Bio-safety, 2 Yuanmingyuan West Road, Beijing, 100193 China; 2 London Research and Development Centre, Agriculture and Agri-Food Canada, 1391 Sandford Street, London, ON N5V 4T3 Canada; 3 Ottawa Research and Development Centre, Agriculture and Agri-Food Canada, 960 Carling Avenue, Ottawa, ON K1A 0C6 Canada; 4 Agassiz Research and Development Centre, Agriculture and Agri-Food Canada, 6947 Highway 7, Agassiz, BC V0M 1A0 Canada; 5 Systematic Entomology Laboratory, USDA-ARS c/o NMNH, Smithsonian Institution, 10th & Constitution Ave NW, MRC 168, Washington, DC 20560 USA; 6 CABI, Rue des Grillons 1, 2800 Delemont, Switzerland; 7 Florida State Collection of Arthropods, Florida Department of Agriculture and Consumer Services, Division of Plant Industry, 1911 SW 34th St., Gainesville, FL 32608 USA

**Keywords:** Egg parasitoid, Biological control, Brown marmorated stink bug, Ecological host range, Fundamental host range

## Abstract

The brown marmorated stink bug, *Halyomorpha halys* (Stål), native to China, Japan, and Korea, has emerged as a harmful invasive pest of a variety of crops in North America and Europe. The Asian egg parasitoid *Trissolcus japonicus* has been identified as the most promising agent for classical biological control of invasive *H. halys* populations. A 4-year study evaluated the fundamental and ecological host ranges of *T. japonicus* as well as its phenology and impact on *H. halys* populations in fruit orchards in its native range in northern China. In laboratory no-choice tests, developmental suitability of eight non-target host species for *T. japonicus* was demonstrated by the successful production of progeny on the majority (>85%) of non-target host species tested. In field-collected, naturally laid egg masses, *T. japonicus* was the most abundant parasitoid associated with *H. halys* and *Dolycoris baccarum*, but was also sporadically found in *Plautia crossota*. Furthermore, it was regularly reared from sentinel egg masses of *Menida violacea*, *Arma chinensis*, and *Carbula eoa*. The only species that did not support development in the laboratory and field was *Cappaea tibialis*. Besides the benefit of having a high impact on *H. halys* populations in Northern China, the risk assessment conducted in the area of origin indicates that native Pentatomidae in North America and Europe could be negatively impacted by *T. japonicus*. Whether the benefits of *T. japonicus* outweigh the possible risks will have to be evaluated based on the outcome of additional host range studies in the two invaded regions.

**Electronic supplementary material:**

The online version of this article (doi:10.1007/s10340-017-0863-y) contains supplementary material, which is available to authorized users.

## Key message

- The Asian egg parasitoid *Trissolcus japonicus* is considered the most promising species for classical biological control of *Halyomorpha halys*
- We investigated the fundamental and ecological host range of *T. japonicus* in northern China to define its host specificity
- *T. japonicus* does successfully develop on Pentatomidae other than its intended target, *H. halys*, under laboratory and field conditions
- Negative impacts on native non-target species must be further studied to determine whether the benefit outweighs the risk if *T. japonicus* is released outside of Asia
- The accidental establishment of *T. japonicus* in North America provides an opportunity to monitor its impacts on non-target species and *H. halys* and to quantify the risks and benefits.

## Introduction

The brown marmorated stink bug, *Halyomorpha halys* (Stål) (Hemiptera: Pentatomidae), native to subtropical and temperate areas in East Asia (China, Japan, and Korea), has emerged as a harmful invasive pest of a variety of crops in North America and Europe (Hoebeke and Carter 2003; Haye et al. 2014a, 2015b). Bioclimatic models further suggest that favourable conditions for establishment of *H. halys* exist in South America, southern Africa, and Oceania (Zhu et al. 2012; Haye et al. 2015b). The development of large pest populations outside its native range has recently generated secondary invasions within Europe and has accelerated the global spread of this pest, primarily through human-mediated activities (Gariepy et al. 2013, 2015).

In Asia, North America, and Europe, *H. halys* is highly polyphagous, feeding on a wide variety of native and exotic host plants, including many economically important field and tree crops, vegetables, ornamentals, herbaceous perennials, shrubs, and forest trees (Lee et al. 2013; Haye et al. 2014b; Martinson et al. 2015; Bergmann et al. 2016). Damage is caused by feeding by the adults and nymphs on fruits, buds, leaves, and stems. In the mid-Atlantic region of the USA, *H. halys* has become one of the most significant pests in apple production, causing >$37 million in losses in 2010 (United States Apple Association 2010). In recently invaded areas in Europe and Eurasia, including northern Italy and western Georgia, severe damage has been observed in pear (*Pyrus communis* L.) and hazelnut (*Corylus avellana* L.) orchards since the arrival of *H. halys* (Bariselli et al. 2016).

Currently, chemical control is the most widely used tactic for managing *H. halys* in the Northeast of the USA; however, the most effective insecticides are generally broad spectrum in their activity, disrupting natural enemies of other pests, and thus negatively interfering with existing IPM programs (Leskey et al. 2012a, b). More environmentally friendly and self-sustaining control measures, such as conservation of existing natural enemies in invaded areas, introduction of specialized egg parasitoids from Asia for classical biological control, and the use of indigenous parasitoids for augmentative control, are currently being investigated and may become important management tools in the near future for area-wide control of *H. halys*.

In its native range, eggs of *H. halys* are attacked by a complex of species in the genera *Trissolcus*, *Telenomus*, *Ooencyrtus* (Platygastridae), and *Anastatus* (Eupelmidae), whereas nymphs and adults are rarely parasitized (Arakawa and Namura 2002; Yang et al. 2009; Lee et al. 2013). Within the egg parasitoid guild, *Trissolcus japonicus* (Ashmead) (syn. *Trissolcus halyomorphae* Yang; Talamas et al. 2013) has been identified as most promising for classical biological control of invasive *H. halys* populations, causing high levels of parasitism in China (Yang et al. 2009). Single *T. japonicus* females lay 42 eggs on average and prefer to oviposit in eggs no older than 1–3 days (Qiu et al. 2007). The wasp reproduces much more rapidly than its host *H. halys*, completing development from egg to adult within 10.5 and 7.3 days at 25 and 30 °C, respectively. The number of degree days required for completion of development is 132.5 DD, suggesting that *T. japonicus* could potentially have up to 10 generations per year in southern China (Qiu et al. 2007; Yang et al. 2009). In addition, offspring are heavily female biased, and the parasitoids overwinter as adults, which are chill-intolerant, but appear more cold-tolerant than their hosts, *H. halys* (Santacruz et al. 2017). Besides these desirable attributes for potential biological control agents, Qiu (2007) reported that *T. japonicus* was also successfully reared on three other pentatomid species, *Erthesina fullo* (Thunberg), *Dolycoris baccarum* (L.), and *Plautia crossota* (Dallas), in the laboratory, suggesting that it may have a broad host range.

In the past decade, most countries have implemented strict regulatory requirements for the importation and release of natural enemies, with detailed risk assessment and host range studies required as part of a petition process for agent approval (Mason et al. 2013). Current practices include a comparison of field-generated host range data (ecological host range) from the native range of the biocontrol agent with that generated in laboratory studies (fundamental host range) (Van Driesche and Reardon 2004; Bigler et al. 2006; van Lenteren et al. 2006).

Laboratory host specificity tests with North American and European Pentatomidae species are currently being conducted by USDA-ARS, CABI, and other laboratories, respectively, but the ecological host range of *T. japonicus* and its seasonal impact on *H. halys* populations in Asia are still poorly understood. Here, we present results from a 4-year study evaluating the fundamental and ecological host ranges of *T. japonicus* in its native range in northern China and elucidating its phenology and impact on *H. halys* populations in fruit orchards throughout the season. We use these results to predict the ecological host range and possible non-target impacts of *T. japonicus* in areas invaded by *H. halys*. These predictions are particularly relevant due to the recent adventive establishment of *T. japonicus* in North America (Talamas et al. 2015), as this provides the opportunity to validate the predictions of this study with the realized, ‘post arrival’, host range as it manifests over time. However, we cannot exclude the possibility that the *T. japonicus* strains not deliberately released to North America may differ in terms of host specificity from the Chinese strain tested here.

## Materials and methods

### Fundamental host range

#### Rearing of target host species

A colony of *H. halys* was established from adults collected in Beijing and Hebei provinces (Table 1) and maintained at the Joint Laboratory of the Ministry of Agriculture-Centre for Agriculture and Biosciences International (MoA-CABI), Beijing, China, in continuous rearing on a diet of green bean pods (*Phaseolus vulgaris* L.) and fresh ears of corn (*Zea mays* L.) at 25 ± 1 °C, 60–70% RH and 16:8 L:D in gauze cages (60 × 60 × 60 cm). In summer, fresh-cut branches of peach [*Prunus persica* (L.) Batsch] trees were offered as an additional food source and oviposition substrate and changed when needed. Leaves with newly laid eggs were collected from the adult cages daily and maintained under the same conditions in separate nymphal rearing cages.

**Table 1:** Non-target species from the family Pentatomidae selected for host specificity tests with *Trissolcus japonicus*

Test species	Host plant	Selection criteria	Origin of laboratory cultures
Subfamily: Pentatominae			
Tribe: Cappaeini			
*Halyomorpha halys* Stål	*Robinia pseudoacacia* L.; *Prunus persica* (L.) Batch	Target	Langfang (Hebei Province) Changping, Lengquan (both Beijing City)
*Cappaea tibialis* Hsiao and Cheng	*Robinia pseudoacacia* L.	Habitat and host plant overlap, close relatedness	Baiwang Mountain (Beijing City)
*Homalogonia obtusa* (Walker)	*Malus baccata* (L.) Borkh.	Habitat and host plant overlap, close relatedness	Miaofeng Mountain (Beijing City)
Tribe: Carpocorini			
*Dolycoris baccarum* (L.)	Various weeds	Habitat overlap, literature host record	Langfang (Hebei Province) Lengquan (Beijing City)
Tribe: Halyini			
*Erthesina fullo* (Thunberg)	*Paulownia tomentosa* (Thunb.) Steud.	Habitat and host plant overlap, literature host record	Liuzhou (Guangxi Province)
Tribe: Eysarcorini			
*Carbula eoa* (Bergroth)	*Artemisia annua* L.	Habitat overlap	Miaofeng Mountain (Beijing City)
Tribe: Antestiini			
*Plautia crossota* Fabricius	*Morus alba* L.	Habitat and host plant overlap	Lengquan, Shujiatuo (both Beijing City)
Tribe: Menedini			
*Menida violacea* Motschulsky	*Morus alba* L.	Habitat and host plant overlap	Yangtai Mountain (Beijing City)
Subfamily: Asopinae			
Tribe: Asopini			
*Arma chinensis* (Fallou)	(Predatory species)	Beneficial species	Sino-American Biological Control Laboratory culture, Beijing

#### Selection, source, and rearing of non-target host species

The selection of non-target hosts for laboratory testing was based on phylogenetic criteria, accessibility, sympatry of target and non-target species, and information on the parasitoid biology available from the literature (Table 1) (Kuhlmann et al. 2006).

Native non-target stink bugs (Hemiptera: Pentatomidae) were collected from their host plants in the spring of each year from various locations in the Beijing and Guangxi provinces (Table 1). All herbivorous species were fed with beans and corn or their associated host plants (Table 1) and kept under the same conditions as described above. Adults of the predatory species, *Arma chinensis* (Fallou), were obtained from the Sino-American Biological Control Laboratory in Beijing and kept in small 0.5-l plastic containers. Pupae of the Chinese oak moth, *Antheraea pernyi* Guérin-Méneville (Lepidoptera: Saturniidae), were removed from their surrounding cocoons and placed on the gauze lids of each container as food source. Egg masses of each non-target species were collected from rearing cages on a daily basis.

#### Source and rearing of *Trissolcus japonicus*

*Trissolcus japonicus* was reared from sentinel *H. halys* egg masses exposed in a fruit orchard at Lengquan, Beijing Province. Laboratory colonies were maintained in acrylic cages (25 × 25 × 25 cm) at 25 ± 1 °C, 60–70% RH and 16:8 L:D. Parasitoid colonies were fed daily with 10% honey water solution and provided with newly laid egg masses of *H. halys* for oviposition. Parasitized egg masses were moved to a separate cage when parasitoid adults were close to emergence, as indicated by the dark colouration of the egg masses. Newly emerged wasps were kept together for two days to ensure mating had occurred and used for testing when they were three days old. Upon the initial establishment of the laboratory colonies of *T. japonicus*, specimens were sent to the Systematic Entomology Laboratory, USDA-ARS (USA), and their identification was verified by E. Talamas.

#### Laboratory no-choice tests

The no-choice tests were designed to determine whether egg masses of non-target hosts were suitable for parasitoid development. In each experimental set-up, similar numbers of randomly selected, naïve, 2–3-day-old *T. japonicus* females were separately tested simultaneously on egg masses of the target *H. halys* (control) and the non-target species listed in Table 1 (between 4 and 61 replicates per non-target species). All egg masses and parasitoids were taken from the laboratory colonies described above. Each parasitoid female was offered a single, newly laid (0–48 h) egg mass of either the target or non-target species. Egg masses were exposed to single females for 24 h in a Petri- dish experiment (5 cm diameter). A cotton wick saturated in honey water was provided as a food source for the wasps. After 24 h, the wasps were removed and the eggs were reared at 25 ± 1 °C, 60–70% RH and 16:8 L:D light until all parasitoids and/or nymphs had emerged, up to 3 weeks. The number of emerged parasitoids, nymphs and dead eggs (=‘no emergence’) was recorded as well as the sex ratio of the parasitoid offspring.

### Ecological host range

#### Field-collected egg masses

In northern China, surveys for stink bug egg masses were conducted at 12 sites in Beijing and Hebei provinces from 2012 to 2015 to document the egg parasitoid assemblages of *H. halys* and co-occurring non-target Pentatomidae.

Sites included agricultural research stations with a wide variety of trees and shrubs (Langfang, Beijing), fruit orchards with a mix of peach, cherry (*Prunus avium* L.), mulberry (*Morus alba* L. and *M. nigra* L.), and apple (*Malus pumilla* Miller) trees (Haidian, Hengshui, Lengquan, Shengshuyuan, Shujiatuo), or natural forests and parks (Baiwang Mountain, Miaofeng Mountain, Fragrant Hills Park, Xishanlinyu, Xiaojiahe). Sites with high densities of *H. halys* adults (e.g. Langfang) were visited more than once per year. At each site, the underside of leaves of trees and shrubs were carefully inspected for the presence of stink bugs and their egg masses. Leaves with egg masses were removed from their host plants and taken back to the MoA-CABI Joint Laboratory in Beijing, where they were maintained individually in small Petri dishes (5 cm diameter) at 25 ± 1 °C, 60–70% RH and 16:8 L:D until all parasitoids and/or nymphs had emerged, up to 7 weeks. Emerged parasitoids were transferred into ethanol, and subsamples were sent to E. Talamas (Systematic Entomology Laboratory, USDA-ARS, USA) and M. C. Bon (European Biological Control Laboratory, Montpellier, France) for morphological and molecular identification, respectively. Egg masses of non-target species were identified by comparing field-collected egg masses with egg masses from our laboratory colonies of non-target species. If it was not possible to identify an egg mass based on morphological comparison with our laboratory colonies, hatched nymphs were reared to the adult stage for identification. Taxonomic guidance was provided by W. Bu (Nankai University, China).

#### Exposure of sentinel egg masses

As egg masses of Pentatomidae are often well-camouflaged and difficult to locate in the foliage of trees and shrubs, it can be challenging to find sufficient numbers in the field. To supplement the information provided by naturally laid, field-collected egg masses, sentinel egg masses from laboratory-reared non-target species were exposed at various sites to determine whether non-target egg masses would be attacked by *T. japonicus* under field conditions.

In 2013, sentinel eggs of *H. halys*, *D. baccarum*, and *P. crossota* were exposed weekly in two orchards near Lengquan (N40°02′06″; E116°12′41″) and Shengshuyuan (N40°03′07″; E116°06′44″), both located in the suburbs of Beijing City. *Halyomorpha halys* eggs served as a control for the presence of *T. japonicus* at the selected sites. At the mixed orchard near Lengquan, peach leaves with newly laid egg masses (collected the same day or stored at 10 °C for maximum 48 h to prevent development) were stapled on the underside of leaves of randomly selected peach, white mulberry (*M. alba*) or jujube trees (*Ziziphus jujube* Mill.) in the interior of the orchard. At Shengshuyuan, egg masses were exclusively exposed on mulberry trees. The number of egg masses exposed per week and per species varied with availability, ranging from 4 to 10. Egg exposure at Lengquan and Shengshuyuan started in early May and early June, respectively, and lasted until early September.

In 2014, six sites located in the suburbs of Beijing City were selected for sentinel egg exposure, including three orchards [Lengquan (peach/cherry/mulberry orchard), Sujiatuo (peach/cherry orchard; N40°03′58″; E116°06′46″), Yantai mountain (peach orchard; N40°04′15.84″; E116°04′53.12)], and three natural forest sites in the western mountains near Beijing [Beianhe village (N40°04′8.5.52″; E116°04′54.43″), Baiwang Mountain (N40°01′53″; E 116°15′32″), Fragrant Hills (N40°03′58.51″; E116°03′0.18″)]. Egg masses of *H. halys*, *D. baccarum*, *P. crossota, Menida violacea* Motschulsky, and *A. chinensis* were exposed every two weeks between May and August at each of the selected sites. At Fragrant Hills, egg masses were exposed on smoke trees [*Cotinus coggygria* (Scopoli)], whereas at all other sites, egg masses were stapled to peach trees.

Exposed egg masses were marked with coloured flagging tape and retrieved after five days. Egg masses were kept separately in small Petri dishes at 25 ± 1 °C, 60–70% RH and 16:8 L:D until all parasitoids and/or nymphs had emerged.

#### Statistical analysis

In small arena no-choice tests, the proportion of females successfully producing offspring (=suitability) and the proportion of male and female offspring (sex ratio) were compared among treatments (=host species) using a Pearson Chi-Square test. To compare parasitism levels, only egg masses from which parasitoids had emerged were included in the analysis. Parasitism levels (=proportion of eggs producing offspring within an egg mass) were calculated by the number of emerged parasitoids divided by the total number of eggs in the mass. Egg mortality was defined as the proportion of eggs from which neither nymphs nor parasitoids had emerged. The average successful development and the average egg mortality in the control (*H. halys*) and the different treatments (non-target hosts) were compared pairwise, using Mann–Whitney U tests. All statistical analyses were carried out with the SPSS^®^ 20.0 software package (IBM Corp. 2013).

## Results

### Fundamental host range

**Fig. 1:**
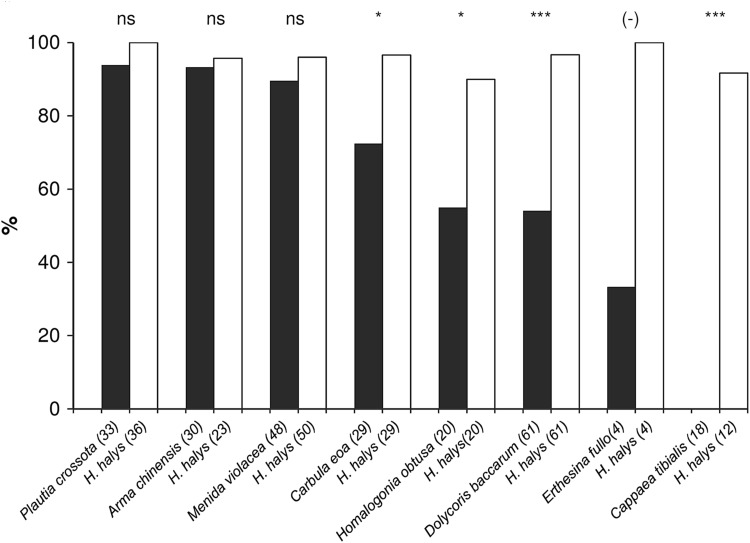
Percentage of *T. japonicus* females successfully parasitizing egg masses of *Halyomorpha halys* (*white bars*) and non-target hosts (*black bars*) in small arena no-choice tests. The number of tested females is given in *brackets* for each species. *Bars* marked with *asterisks* indicate a significant difference between groups (Pearson Chi-Square test: *p < 0.05; **p < 0.01; ***p < 0.001; *ns* not significantly different). Due to the low number of replicates, *E. fullo* was not included in the analysis (–)

In total, seven out of eight non-target hosts were accepted and suitable for parasitoid development (Fig. 1). Levels of suitability (=proportion of females successfully producing offspring from >50% of eggs in an egg mass) were not significantly different for three non-target species, *P. crossota, M. violacea* and *A. chinensis,* than for the target host *H. halys*. Suitability of the target *H. halys* (controls) was generally high, varying between 90 and 100% (average 95.8%). In contrast to the *H. halys* controls, rates of suitability of *Carbula eoa* (Bergroth) (72.4%) (Pearson Chi-Square test, *χ* ^2^ = 6.444, *p* = 0.011), *Homologonia obtusa* (Walker) (55.0%) (*χ* ^2^ = 6.144, *p* = 0.013), and *D. baccarum* (54.1%) (*χ* ^2^ = 29.881, *p* < 0.0001) were significantly lower. The only species from which no *T. japonicus* offspring developed was the closely related *C. tibialis*, which belongs to the same tribe (Cappaeini) as *H. halys*. For *E. fullo*, test data are preliminary as only four females could be tested, and only one successfully produced offspring.

**Fig. 2:**
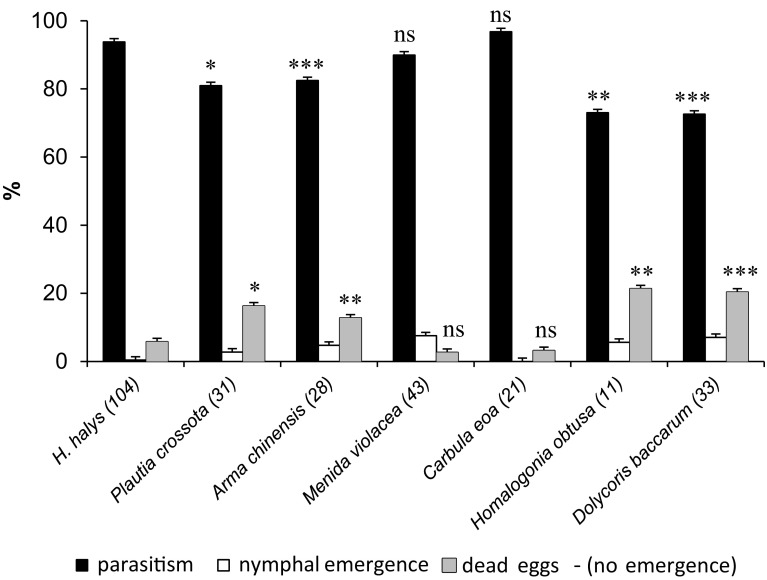
Mean (±SE) proportion of successfully parasitized eggs (=parasitoid emergence) and egg mortality (no emergence of nymphs or parasitoids) within individual egg masses in small arena no-choice tests. The number of replicates is given in *brackets* for each species. *Bars* marked with *asterisks* indicate a significant difference between control (*H. halys*) and treatments (non-target species) groups, (Mann–Whitney test: *p* < 0.05; **p < 0.01; ***p < 0.001; *ns* not significantly different)

Parasitism levels (=proportion of eggs producing offspring within an egg mass) were generally high (>70%) for all species tested (Fig. 2), but not significantly different between *H. halys* (93.8%) and the two non-target hosts *M. violacea* (89.9%) and *C. eoa* (96.8%) (Mann–Whitney tests, *Z* = −1.192; *Z* = −1.343, both *p* > 0.05). In contrast, in *P. crossota* (*Z* = −2.370, *p* = 0.018), *A. chinensis* (*Z* = −3.569, *p* ≤ 0.001), *H. obtusa* (*Z* = −2.862, *p* = 0.004), and *D. baccarum* (*Z* = −4.635, *p* ≤ 0.001), parasitism levels were significantly lower than in the *H. halys* controls, and egg mortality (=no emergence of nymphs or parasitoids) for these four species was significantly higher (Fig. 2).

**Fig. 3:**
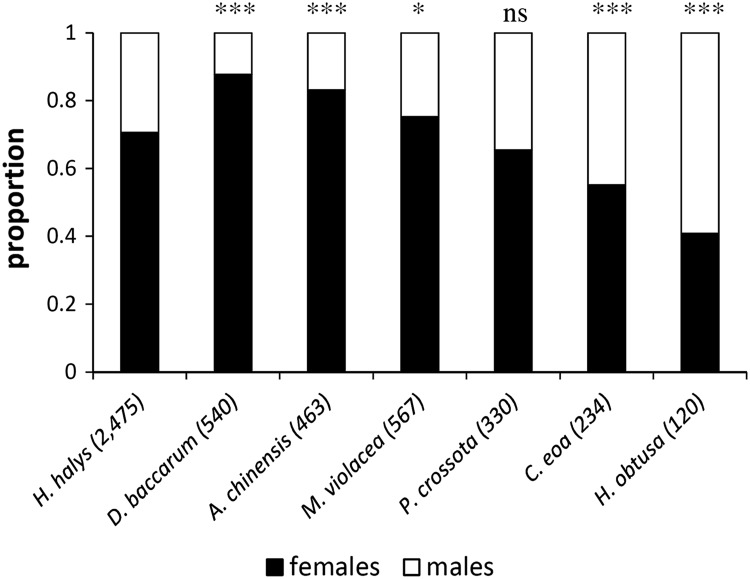
Sex ratio of offspring reared from *H. halys* and six non-target hosts (in *brackets*: no. of individuals emerged); *bars* marked with *asterisks* indicate a significant difference between control (*H. halys*) and treatments (non-target species) groups (Pearson Chi-Square test: *p* < 0.05; **p < 0.01; ***p < 0.001; *ns* not significantly different)

The proportion of *T. japonicus* females emerging from *H. halys* eggs (control) was 70.6%, whereas for *C. eoa* (55.1%, *χ* ^2^ = 23.989, *p* < 0.0001) and *H. obtusa* (40.8%, *χ* ^2^ = 47.534, *p* < 0.0001), it was significantly lower (Fig. 3). From eggs of *M. violacea* (75.3%, *χ* ^2^ = 5.046, *p* = 0.025), *A. chinenis* (83.1%, *χ* ^2^ = 30.944, *p* < 0.0001), and *D. baccarum* (87.8%, *χ* ^2^ = 67.536, *p* < 0.0001), significantly more females emerged. The proportion of females emerging from *P. crossota* eggs was not significantly different from the control (65.5%, *χ* ^2^ = 3.650, *p* = 0.056).

### Ecological host range

#### Field-collected egg masses

**Fig. 4:**
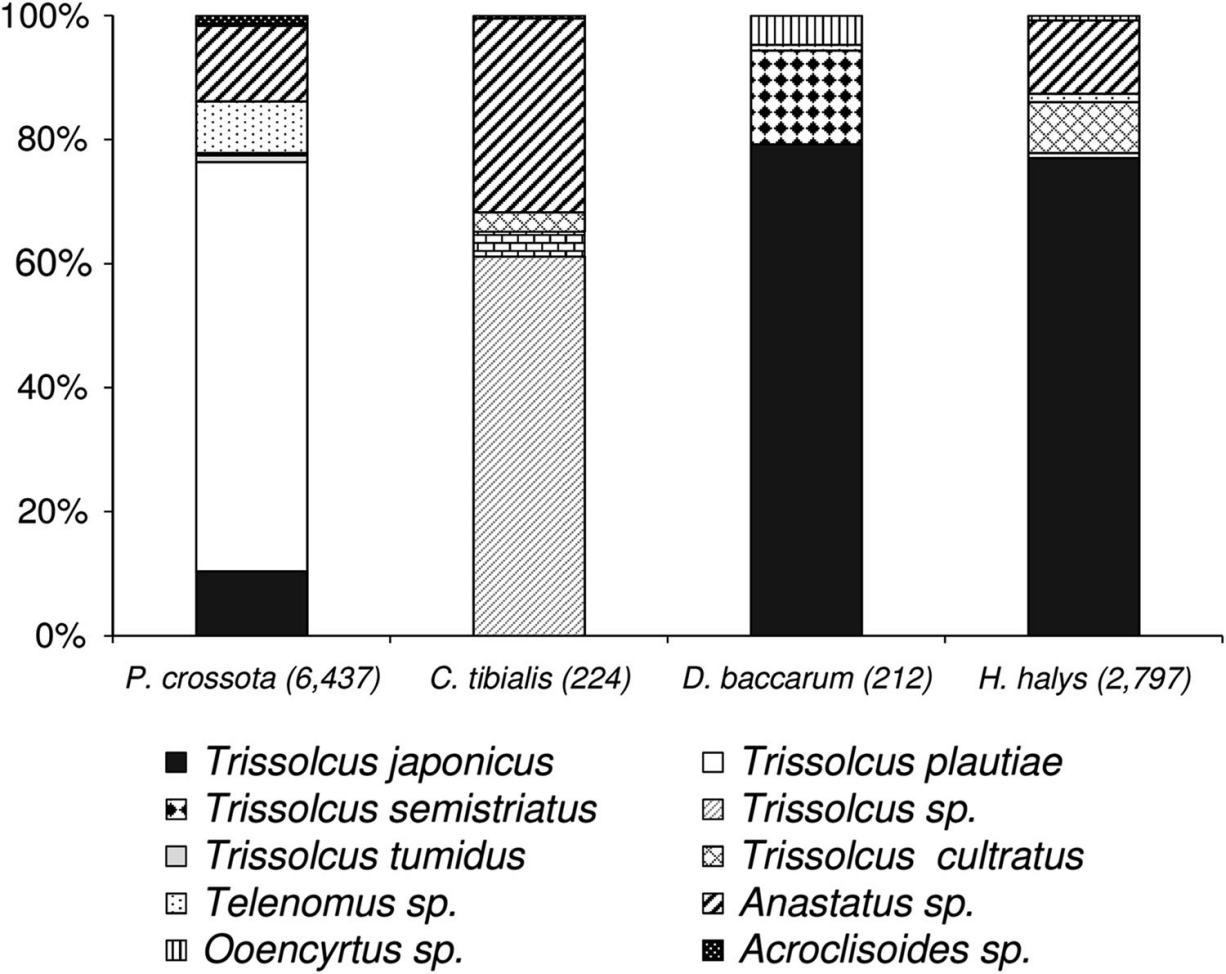
Species composition of parasitoids reared from field-collected egg masses of *Halymorpha halys* and three non-target species (in *brackets*: no. of emerged parasitoids). For details, see supplementary Table 1

In total, 186 out of 236 *H. halys* naturally laid egg masses (=5944 eggs) were parasitized (78.8%) (Supplementary Table 1). From those egg masses, 2799 parasitoids were reared. Consistent with previous studies (Qiu et al. 2007; Yang et al. 2009), our field collections confirmed that *T. japonicus* is the most abundant egg parasitoid of *H. halys* in northern China (proportion: 77.2%, *N* = 2799), followed by *Anastatus* sp. (11.8%) and *Trissolcus cultratus* (8.1%) (Fig. 4; Supplementary Table 1). Other species such as *Trissolcus plautiae* (0.8%), *Telenomus* sp. (1.3%), and *Ooencyrtus* sp. (0.8%) were reared sporadically. Parasitized egg masses were found on 11 different host plants, but most egg masses (46.5%) were collected from black locust (*Robinia pseudoacacia* L.).

The diversity and abundance of non-target Pentatomidae in the Beijing suburbs was generally low, and thus, collecting sufficient numbers of egg masses was challenging. In total, 995 egg masses belonging to four non-target species were collected, from which a total of 6879 parasitoids were reared (supplementary Table 1). The only abundant species in fruit orchards was *P. crossota*, a common species on mulberry trees. In total, parasitoids emerged from 77.9% of the *P. crossota* egg masses (*n* = 891). The dominant egg parasitoids of *P. crossota* were *T. plautiae* (66.0%), followed by *Anastatus* sp. (12.2%) and *T. japonicus* (10.4%) (Fig. 4; Supplementary Table 1). At Lengquan, where eggs of *P. crossota* were collected continuously in 2013, the proportion of *T. japonicus* increased from 0.8% in May (*n* = 1223) to 31.6% in July (*n* = 316), the end of the egg-laying period of *P. crossota* (Supplementary Table 1). Furthermore, *T. japonicus* comprised 79.3% of the egg parasitoid species of *D. baccarum* (*n* = 212), whereas the proportion of *T. semistriatus* was 15.1% (Fig. 4). Egg masses of *C. tibialis* (*n* = 71) were only found on black locust trees at Baiwang Mountain. There were no *T. japonicus* among the parasitoids emerging from eggs of *C. tibialis* (*n* = 1108), which were primarily parasitized by an undescribed *Trissolcus* sp. (61.2%) and *Anastatus* sp. (31.3%) (Fig. 4). *Trissolcus tumidus* and the rare hyperparasitoid *Acroclisoides* sp. (Hymenoptera: Pteromalidae) were only sporadically reared. Overall parasitism of *C. tibialis* was 20.2%. Only two egg masses of *A. chinensis* were found, from which six *Anastatus* sp. were reared.

#### Exposure of sentinel egg masses

**Fig. 5:**
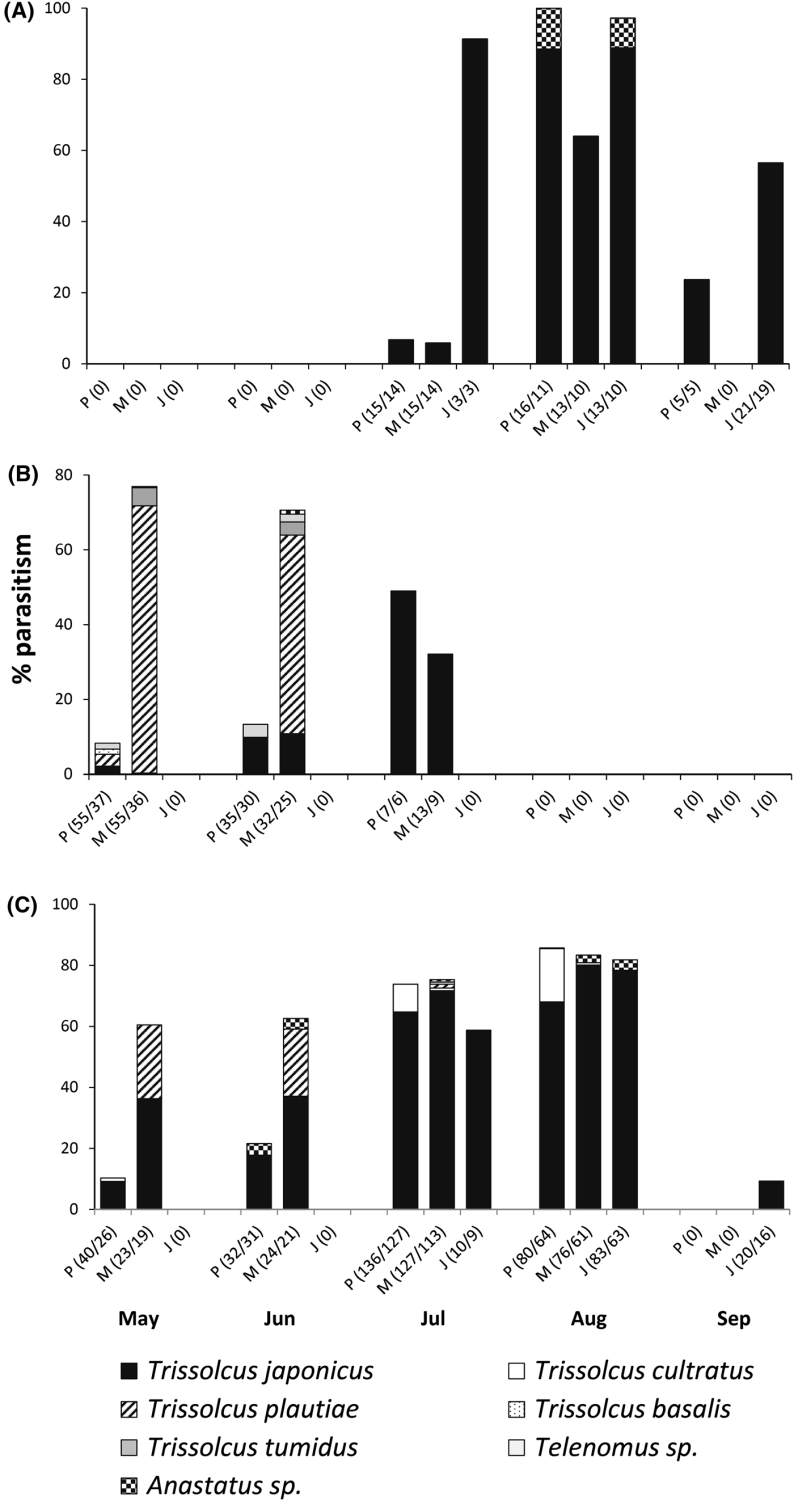
Parasitism of **a** *Dolycoris baccarum*, **b** *Plautia. crossota*, and **c** *Halyomorpha halys* egg masses exposed on three different types of trees (*P* peach; *M* mulberry; *J* jujube tree) at Lengquan in 2013. *Numbers* in *brackets* indicate the number of egg masses exposed/recollected. Exposure on jujube trees was not started before July

In 2013, 289, 250, and 115 *H. halys* egg masses were exposed on peach, mulberry, and jujube trees, respectively, in an orchard near Lengquan. In total, 13,868 eggs were recollected, from which 6767 parasitoids were reared. *Trissolcus japonicus* was by far the most abundant species from host eggs on all three types of trees, whereas the other parasitoid species were of minor importance. Parasitism by *T. japonicus* on peach trees increased from 9.2% in May to 68% in August (Fig. 5c). On mulberry trees, parasitism was higher from the beginning (36.3%), but peak parasitism by *T. japonicus* was also observed in August (80.1%). Remarkably, parasitism by *T. plautiae* was more than 20% in May and June. On jujube trees, parasitism by *T. japonicus* was 58 and 78% in July and August, respectively, and *T. japonicus* still emerged in small numbers from eggs exposed in early September. Simultaneously exposed egg masses of *P. crossota* on mulberry trees were heavily parasitized by *T. plautiae* in the months May and June, whereas eggs exposed on peach trees were far less parasitized (Fig. 5b). In these months, parasitism by *T. japonicus* was low and did not exceed 10%. However, when *T. plautiae* stopped being active in the field in July, exposed *P. crossota* eggs were exclusively parasitized by *T. japonicus*. Egg masses of *D. baccarum* were regularly attacked in the field and the highest parasitism levels were observed in August, ranging from 63 to 88% (Fig. 5a). Remarkably, nearly all parasitoids reared from *D. baccarum* were *T. japonicus*.

**Fig. 6:**
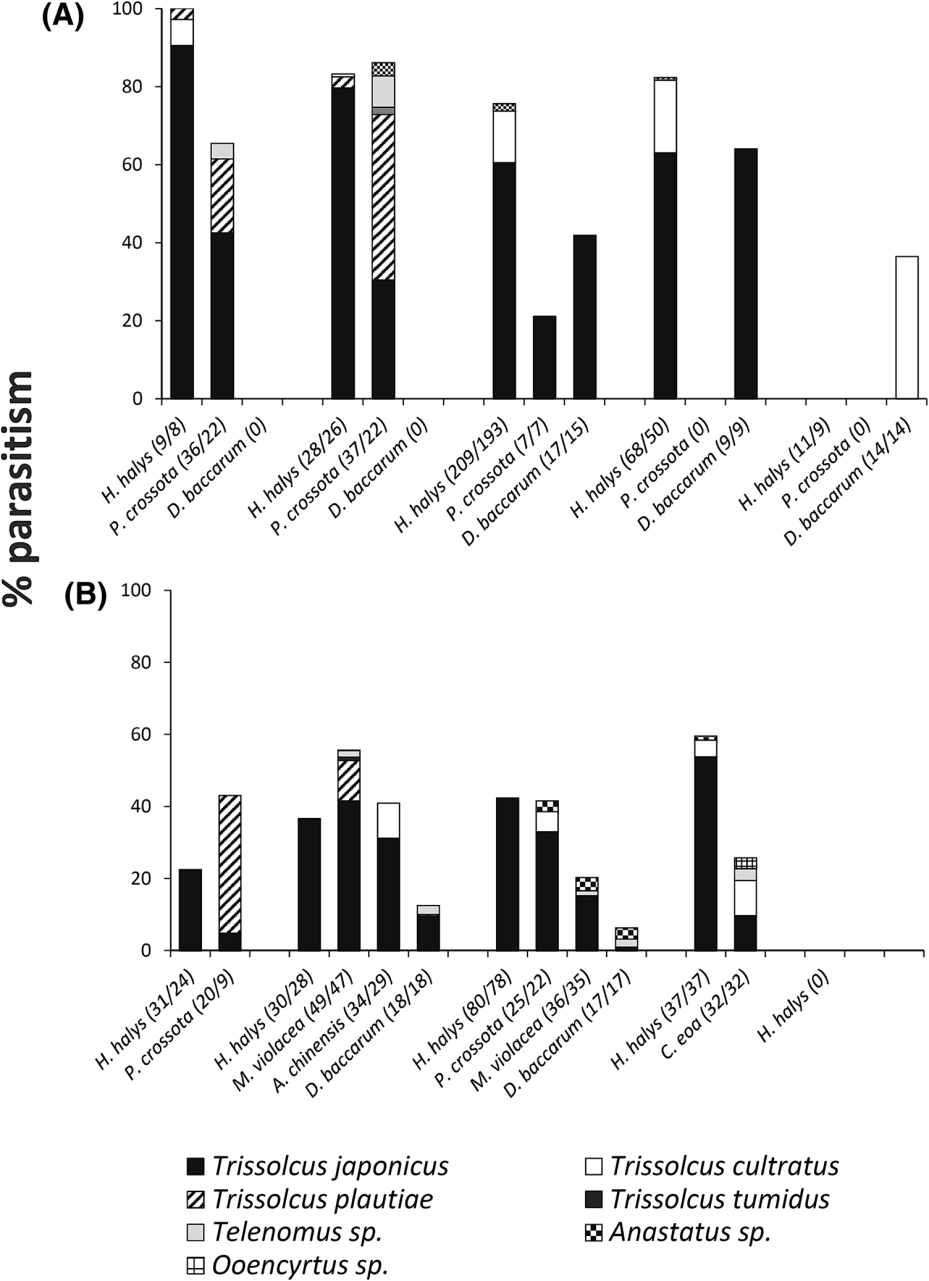
**a** Parasitism of *H. halys, P. fimbriata*, and *D. baccarum* egg masses exposed on mulberry trees at Shengshuyuan in 2013 and **b** parasitism of *Pentatomidae* egg masses exposed at Lengquan in 2014. Egg masses were exposed on peach trees, except *Plautia crossota* which were exposed on mulberry trees. *Numbers* in *brackets* indicate the number of egg masses exposed/recollected

At Shengshuyuan, where *H. halys* eggs (*n* = 8569) were only exposed on mulberry trees, *T. japonicus* was by far the most abundant species (81.2%), followed by *T. cultratus* (16.4%) (Fig. 6a). Parasitism levels by *T. japonicus* were continuously high throughout the summer, ranging from 64 to 90%. Similar to the Lengquan exposure site, *P. crossota* eggs were mainly parasitized by *T. plautiae*, but parasitism by *T. japonicus* was higher in May (42 vs. 20%) but lower in June (30 vs. 75%). In July and August, exposed eggs of *P. crossota* and *D. baccarum* were exclusively parasitized by *T. japonicus* (Fig. 6a).

In 2014, sentinel egg exposure at Lengquan was continued, but *H. halys* egg masses were only exposed on peach trees (Fig. 6b; Supplementary Table 2). Similar to the previous year, parasitism of *H. halys* was lowest in May (22.5%) and steadily increased until August (59.6%). In the first three months, parasitism was exclusively caused by *T. japonicus*, confirming its importance for the natural control of *H. halys*. However, it was also the dominant parasitoid of *M. violacea*, causing 41 and 15% parasitism in June and July, respectively. Egg masses of the predatory *A. chinensis* were only exposed in June, but parasitism by *T. japonicus* reached 31%. Overall parasitism of sentinel *D. baccarum* eggs was generally lower, and the contribution of *T. japonicus* in June and July was 9 and 1%, respectively. Similar to 2013, sentinel eggs of *P. crossota* exposed on mulberry trees in May 2014 were primarily parasitized by *T. plautiae* and the contribution of *T. japonicus* to overall parasitism was only 5%. However, when *T. plautiae* was no longer present, in July, *T. japonicus* caused 33% parasitism.

**Fig. 7:**
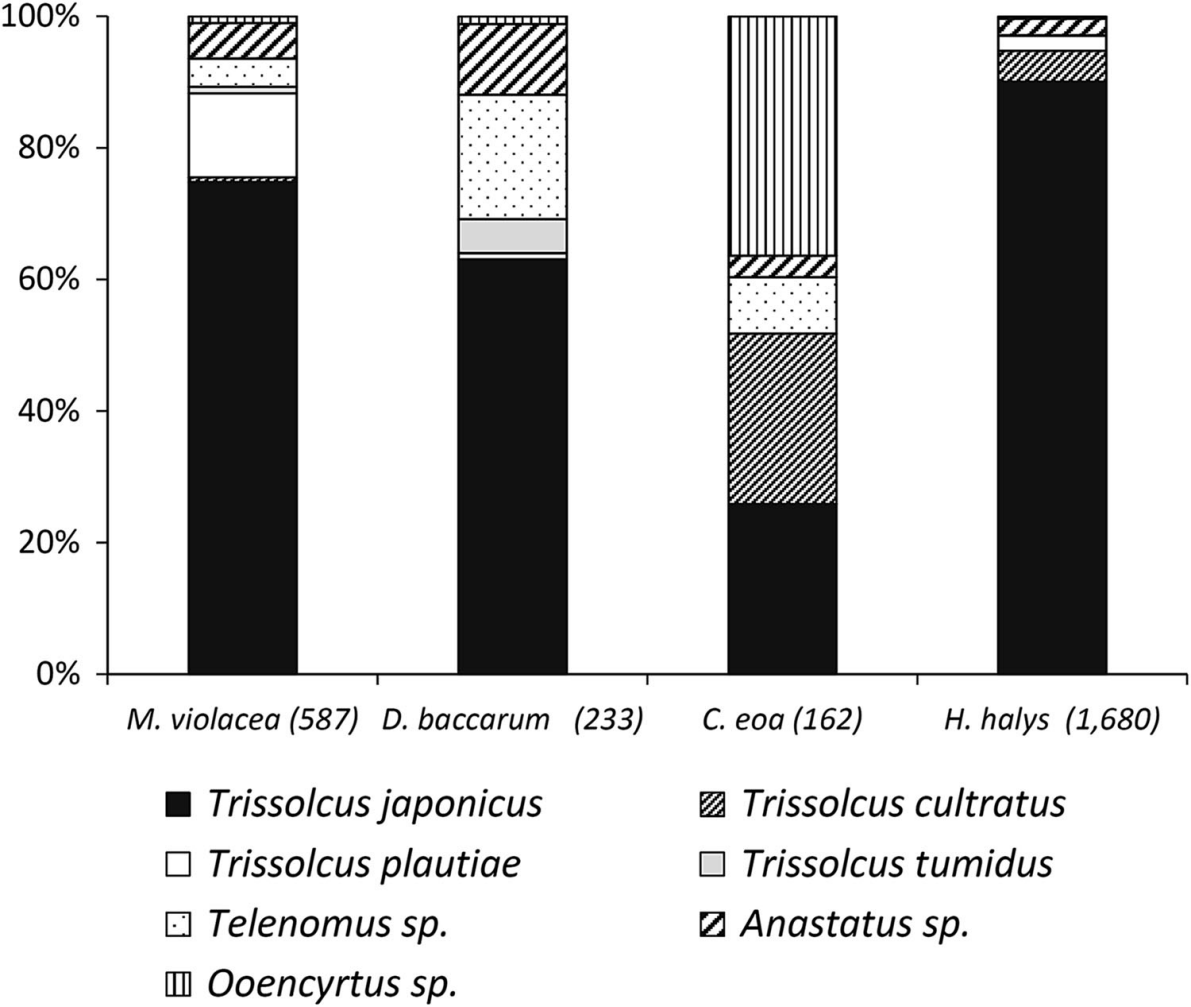
Species composition of parasitoids reared from sentinel egg masses of *Halymorpha halys* and three non-target species exposed at six different sites in 2014 (in *brackets*: no. of emerged parasitoids). For details, see supplementary Table 2

Besides Lengquan, sentinel eggs were exposed at 5 additional sites, but the available number of eggs per month and site was often too low to give conclusive results for each site and species. As such, the data from all sites were pooled in order to provide a comprehensive overview of species composition information (Fig. 7; supplementary Table 2). In total, 1680 parasitoids were reared from sentinel *H. halys* eggs and *T. japonicus* comprised 90.1% of the parasitoid species composition. In addition, *T. japonicus* dominated the parasitoid complex of *M. violacea* and *D. baccarum*, constituting >74 and >63% of the associated parasitoids, respectively. The egg parasitoid complex of *C. eoa* was dominated by *Ooencyrtus* sp. (36.4%), whereas *T. japonicus* and *T. cultratus* were equally abundant (25.9%).

## Discussion

The results of our assessments of the fundamental and ecological host range of *T. japonicus* in Northern China demonstrate that *T. japonicus* does successfully develop on Pentatomidae other than the intended target for classical biological control, *H. halys*, under laboratory and field conditions. On the other hand, the present study confirmed findings of previous studies (Yang et al. 2009), demonstrating the outstanding impact of *T. japonicus* on *H. halys* populations with egg parasitism rates of 50–80% in the months July and August.

In laboratory no-choice tests, developmental suitability of non-target host species for *T. japonicus* was demonstrated by the successful production of progeny on seven out of eight tested non-target host species. Despite the phylogenetic diversity represented in the non-target test list (seven tribes belonging to two subfamilies), *T. japonicus* was able to successfully exploit representatives from each of these tribes. In fact, the suitability for parasitoid development of *A. chinensis*, a predatory stinkbug in the subfamily Asopinae, was not significantly different than that of the target host, *H. halys*, suggesting that *T. japonicus* may have a very broad host range within the family Pentatomidae. Interestingly, closely related species within the same tribe were either not suitable (e.g. *C. tibialis*) or significantly less suitable than *H. halys* (e.g. *H. obtusa*) in terms of the successful production of offspring. This suggests that factors other than phylogenetic relatedness influence the suitability of a host egg for *T. japonicus*. Furthermore, in addition to host acceptance by a parasitoid, behavioural tests are equally important as suitability tests when assessing host specificity (Duan and Messing 1997), and as such, additional studies are recommended to determine whether there are potential behavioural barriers to parasitism that may exist and preclude excessive parasitism in the field.

Although laboratory host range can serve as a predictor of non-target parasitism in the field (Barratt et al. 1997), no-choice laboratory conditions often overestimate the likely host range of a biological control agent (Haye et al. 2005). As such, the ecological host range of *T*. *japonicus* was evaluated to determine the occurrence and level of parasitism of non-target hosts in field conditions. Between 2012 and 2015, a total of 1231 naturally laid, field-collected egg masses belonging to five different pentatomid species were collected and yielded ten different parasitoid species. *Trissolcus japonicus* was the most abundant parasitoid associated with two of these pentatomid species, *H. halys* and *D. baccarum*, but was also sporadically found in *P. crossota* (~10% of the parasitoid species composition). This is consistent with the fundamental host range results, which demonstrated that these hosts are physiologically suitable for development and result in a high level of parasitoid emergence (>70%). Also consistent with the results of the fundamental host range test, *T. japonicus* was not reared from *C. tibialis* egg masses collected in the field, which supports the laboratory no-choice test which showed that this host is not suitable (either physiologically or behaviourally) for development.

Naturally laid, field-collected egg masses certainly provide the most reliable insights into the host selection behaviour of *T. japonicus* in field conditions, but the value of those data is often limited by the sample size. In the present study, the exposure of sentinel target and non-target egg masses at different sites during two field seasons was used as an alternative method to complement the ecological host range data. Similar to field-collected egg masses, *T. japonicus* was the dominant parasitoid associated with sentinel egg masses of *H. halys, D. baccarum*, and *M. violacea* (not represented in the field-collected egg masses). Although *C. eoa* sentinel egg masses yielded *T. japonicus* (26% of the parasitoid species composition), it was not the dominant parasitoid species in this host. Interestingly, the proportion of *T. japonicus* from *H. halys* eggs was >90%, whereas from the non-targets, it was between 26 and 75%, suggesting that these non-target hosts are less preferred. However, egg mortality was also fairly high (23–54%) and thus the presence of *T. japonicus* may be underestimated due to host mortality making it difficult to determine whether the lower prevalence of *T. japonicus* in non-target hosts was due to reduced levels of attack or reduced suitability (resulting in host and parasitoid mortality). The fundamental host range experiments demonstrated that emergence of *T. japonicus* from *M. violacea* and *C. eoa* eggs was not significantly different than from those of *H. halys*. But, in the case of *C. eoa*, the proportion of *T. japonicus* females successfully producing progeny (i.e. indicating developmental suitability) was slightly less than those from *H. halys*. Additional laboratory studies addressing attack and suitability as well as fitness of *T. japonicus* on different non-target hosts may help fill in these gaps. Incorporating molecular tools to detect parasitoid DNA within the unhatched egg may also shed some light on the source of host mortality in field-collected egg masses (Gariepy et al. 2014).

It is important to note that the non-target sentinels were exposed in habitats and on host plants where *H. halys* naturally occurs in high numbers (e.g. peach orchards), which may increase the frequency and likelihood of encounters with *T. japonicus*. The cues used by *T. japonicus* to locate their hosts are still poorly understood. Colazza et al. (2009) demonstrated that in a similar system, *Trissolcus basalis* (Wollaston) perceived chemical footprints left behind by females of its host, *Nezara viridula* (L.), on the plant surface as contact kairomones. If *T. japonicus* uses a similar host finding mechanism, and if chemical foot prints of naturally occurring *H. halys* females were already present on trees prior to the exposure of sentinel eggs, then this may explain the increased incidence and dominance of *T. japonicus* in the parasitoid species composition in sentinel egg masses versus naturally laid, field-collected non-target egg masses. Future non-target studies should therefore focus on the chemical ecology of *T. japonicus*, particularly on the attractiveness of chemical traces of non-target versus target species.

Remarkably, in naturally laid, field-collected egg masses each pentatomid host species had a distinct, dominant parasitoid species: *P. crossota* was predominantly attacked by *T. plautiae*; *C. tibialis* was predominantly attacked by *T. tumidus*; *H. halys* and *D. baccarum* were predominatly attacked by *T. japonicus*. This suggests the potential for resource partitioning in *Trissolcus* species to minimize in-host competition. This is supported by the observation of seasonal changes in the parasitoid species composition in *P. crossota* sentinel egg masses, wherein *T. plautiae* is the dominant parasitoid species in May and June, and *T. japonicus* only appears as the dominant parasitoid in this host in July, when *T. plautiae* is no longer present (Figs. 5b, 6a, b). However, this was not observed in the other non-target hosts, where sentinel eggs masses were predominantly attacked by *T. japonicus*. As previously mentioned, the dominance of *T. japonicus* in these non-targets may be due to the fact that they were exposed in habitats and on host plants where high densities of *H. halys* can be found and therefore may provide an overestimation of the risk to these non-target hosts. Additional studies exposing sentinel egg masses of both the target and non-target hosts in habitats/host plants where *H. halys* is not common would provide additional insight regarding attack levels and parasitoid species composition.

Although there is clear evidence that *T. japonicus* can attack and develop in a variety of non-target Pentatomidae, the population-level impacts remain unclear. However, the concern regarding the impact of *T*. *japonicus* on rare and beneficial pentatomid species is valid. Populations of the endemic Hawaiian pentatomid genera *Coleotichus* and *Oechalia* declined significantly following the introduction of *Trissolcus basalis* for biological control of the invasive southern green stink bug, *Nezara viridula* (Howarth 1991). Laboratory studies on native species of concern should help predict the impact of *T. japonicus* on sensitive, rare, and beneficial species in the area(s) of proposed introduction. Our research suggests that there may be some level of habitat and/or resource partitioning in the field that results in lower levels of parasitism on non-target species versus the target species, *H. halys*. Gariepy and Roitberg (2013) discussed the potential of using mathematical models to assess the potential non-target risks associated with biological control agents. Additional studies using mathematical models that incorporate the data from fundamental and ecological host range studies may be useful to predict the outcome of non-target parasitism and provide a more detailed assessment of population-level impacts of *T. japonicus* in the area of origin and in the areas of proposed introduction.

Non-target impacts may also include those affecting parasitoid species native to the area of introduction. Abram et al. (2014) discovered that egg masses of *H. halys* were attacked by *Telenomus podisi* Ashmead (Hymenoptera: Scelionidae) an egg parasitoid of *Podisus maculiventris* (Say) (Hemiptera: Pentatomidae). However, *T. podisi* adult emergence from *H. halys* eggs was nil compared to >98% for its natural host. Thus, *H. halys* was proposed to be a potential ‘evolutionary sink’ for egg parasitoids of native North American pentatomids, suggesting that parasitoid populations of native pentatomids could decline. Similarly, a recent study discovered that European strains of *Trissolcus cultratus* (Mayr) could not successfully develop on fresh *H. halys* eggs, whereas Chinese strains could (Haye et al. 2015a). However, it was later demonstrated that the European *T. cultratus* strain could emerge from *H. halys* eggs parasitized by *T. japonicus* when the latter species reaches the mature larval stage (Konopka et al. 2017), potentially reducing the impact of the evolutionary trap posed by *H. halys*.

As discussed by Duan and Messing (1997), a better understanding of the predictability of such ecological interactions can only be accomplished by increasing our knowledge of the behavioural ecology and population dynamics of the exotic biological control agent in question. The results of this study provide a piece of this puzzle and have important implications for the impact of *T. japonicus* in North America. Assessment of host range and attack rates in the area of origin indicates that *T. japonicus* may negatively impact a number of the native North American Pentatomidae species (>220 spp. in North America Froeschner 1988). However, the natural enemy ‘sink’ for native parasitoid species proposed by Abram et al. (2014) could have a positive effect on populations of native pentatomids through partial ‘enemy release’.

The discovery that *T. japonicus* has established adventively in the eastern and western USA (Talamas et al. 2015; Milnes et al. 2016) provides an opportunity to not only track its establishment and spread but also to assess impact on non-target Pentatomidae. Retrospective case studies on exotic biological control agents can provide information regarding the environmental effects of an agent and can be used to refine tests and protocols for predicting host specificity (Follet et al. 2000). In the case of *T. japonicus*, monitoring should focus on natural and urban park-type habitats where a diversity of host plants, Pentatomidae and parasitoid species, occurs (see Talamas et al. 2015; Cornelius et al. 2016). These field studies in the invaded regions in North America would test the pre-release predictions arising from the physiological and ecological host range studies conducted in the native range. These data will help to determine whether the introduction of *T. japonicus* into new areas invaded by *H. halys* is ‘risky, or not’.

## Author contributions

TH, PM, TG, and DG conceived research. JZ, FZ, and TH conducted the experiments. TH, TG, and PM wrote the manuscript. ET identified the egg parasitoids. All authors read and approved the manuscript.

## Electronic supplementary material

Below is the link to the electronic supplementary material.

Supplementary Table 1: Species composition of parasitoids reared from field collected egg masses of *Halymorpha halys* and four non-target species (PDF 12 kb)

Supplementary Table 2: Species composition of parasitoids reared from sentinel egg masses of *Halymorpha halys* and three non-target species exposed at various sites in 2014 (PDF 35 kb)
