# Supplementary material for: Seasonal parasitism and host specificity of Trissolcus japonicus in northern China
Source: J Pest Sci (2004). 2017 Apr 18;90(4):1127–41. doi: 10.1007/s10340-017-0863-y (PMC5544787; doi:10.1007/s10340-017-0863-y)
Supplement: Supplementary file 1 — Supplementary Table 1: Species composition of parasitoids reared from field collected egg masses of Halymorpha halys and four non-target species (PDF 12 kb) [file 10340_2017_863_MOESM1_ESM.pdf]

Host	Location	Collection period	host plants	# egg masses (#parasit.)	# eggs	# nymphs emerg.	# dead eggs (no emerg.)	# parasitoids emerg.	*Trissolcus japonicus*	*Trissolcus cultratus*	*Trissolcus plautiae*	*Trissolcus semistriatus*	*Trissolcus sp.*	*Trissolcus tumidus*	*Telenomus* sp.	*Anastatus* sp.	*Ooencyrtus* sp.	*Acroclisoides* sp.
H. halys	Langfang	Jul-12	*Robinia pseudoacacia*	17 (15)	457	117	155	185	177	0	0	0	0	0	0	8	0	0
			Zizyphus jujuba	6 (5)	167	51	54	62	62	0	0	0	0	0	0	0	0	0
			Pyrus sorotina	2 (2)	56	24	8	24	11	0	0	0	0	0	0	13	0	0
			Boehmeria siamensis	4 (4)	91	0	35	56	40	0	0	0	0	0	0	16	0	0
	Haidian	Jul-12	*Pyrus betulaefolia*	6 (6)	145	58	34	53	32	0	0	0	0	0	0	21	**0**	0
	Hengshui	Jul-12	*Ulmus pumila*	2 (1)	57	26	13	18	0	18	0	0	0	0	0	0	**0**	0
			Pyrus sorotina	1 (1)	14	0	7	7	7	0	0	0	0	0	0	0	**0**	0
	Fragrant hill	Jul-12	*Cotinus coggygria*	5 (2)	95	42	41	12	12	0	0	0	0	0	0	0	**0**	0
	Beijing	Jul-12	*Robinia pseudoacacia*	3 (2)	84	28	30	26	26	0	0	0	0	0	0	0	0	0
	Lengquan	May-13	*Prunus persica*	4 (3)	113	26	30	57	39	18	0	0	0	0	0	0	0	0
	Shengshuyuan	Jun/Jul-13	*Prunus persica*	3 (3)	84	0	43	41	41	0	0	0	0	0	0	0	0	0
	Shengshuyuan	May/Jun-13	*Morus alba*	9 (9)	232	6	105	121	92	0	0	0	0	0	4	25	0	0
	Langfang	Jul-13	*Robinia pseudoacacia*	40 (31)	1,096	207	254	635	470	150	0	0	0	0	11	4	0	0
	Baiwang Mt.	Aug/Sep-14	*Robinia pseudoacacia*	34 (21)	699	61	275	363	206	15	0	0	0	0	0	140	2	0
	Fragrant Hills	Aug-14	*Robinia pseudoacacia*	2 (0)	19	0	19	0	0	0	0	0	0	0	0	0	0	0
			Cotinus coggygria	1 (0)	14	0	14	0	0	0	0	0	0	0	0	0	0	0
	Lengquan	May/Jul-14	*Morus alba*	2 (1)	52	26	5	21	0	0	21	0	0	0	0	0	0	0
			Prunus persica	4 (2)	101	45	32	24	24	0	0	0	0	0	0	0	0	0
			Prunus avium	17 (13)	458	25	214	219	171	0	0	0	0	0	9	39	0	0
			Zizyphus jujuba	6 (5)	149	22	36	91	60	0	0	0	0	0	0	31	0	0
	Miaofeng Mt.	Aug-14	*Robinia pseudoacacia*	1 (1)	27	8	12	7	0	0	0	0	0	0	0	7	0	0
	Sujiatuo village	Jul-14	*Prunus avium*	1 (0)	28	28	0	0	0	0	0	0	0	0	0	0	0	0
	Xishanlinyu	Aug-14	*Robinia pseudoacacia*	4 (3)	74	0	51	23	11	0	0	0	0	0	0	7	5	0
	Xiaojiahe	Aug-14	*Robinia pseudoacacia*	1 (0)	22	0	22	0	0	0	0	0	0	0	0	0	0	0
	Baiwang Mt.	May/Jul-15	*Robinia pseudoacacia*	19 (13)	493	151	239	103	92	0	0	0	0	0	0	11	0	0
	Langfang	Jul-15	*Zea mais*	40 (40)	1,062	41	394	627	588	15	0	0	0	0	14	7	3	0
			Morus nigra	2 (2)	55	16	15	24	0	12	0	0	0	0	0	0	12	0
			total	**236 (185)**	**5944**	**1008**	**2137**	**2799**	**2161**	**228**	**21**	**0**	**0**	**0**	**38**	**329**	**22**	**0**
								proportion (%)	77.2	8.1	0.8	0	0	0	1.3	11.8	0.8	0
P. crossota	Lengquan	May-13	*Morus alba*	152 (136)	1,855	205	427	1223	10	0	1122	0	0	30	60	1	0	0
		Jun-13	*Morus alba*	150 (114)	2,088	150	797	1141	105	0	758	0	0	30	29	175	8	36
		Jul-13	*Morus alba*	55 (44)	720	14	390	316	100	0	34	0	0	0	3	157	0	22
	Shengshuyuan	May-13	*Morus alba*	50 (40)	731	131	236	382	132	9	190	0	0	1	41	9	0	0
		Jun-13	*Morus alba*	283 (233)	3,975	298	1,689	1988	272	1	995	0	0	9	382	308	9	12
		Jul-13	*Morus alba*	42 (24)	509	0	418	91	0	0	1	0	0	0	2	84	0	4
	Lengquan	May-14	*Morus alba*	38 (35)	559	40	74	445	15	0	415	0	0	0	8	1	6	0
	Sujiatuo village	May-14	*Morus alba*	88 (56)	1270	363	328	653	9	0	636	0	0	0	8	0	0	0
	Yangtai Mt.	Jun/Jul-15	*Morus alba*	14 (5)	164	20	41	103	4	0	64	0	0	0	0	35	0	0
	Lengquan	Jun/Jul-15	*Morus alba*	14 (4)	155	14	79	65	10	0	33	0	0	0	1	13	0	8
	Baiwang Mt.	May-15	*R. pseudoacacia*	5 (3)	73	33	10	30	13	14	0	0	0	0	0	3	0	0
			total	**891 (694)**	**11,707**	**1268**	**4359**	**6,437**	**670**	**24**	**4248**	**0**	**0**	**70**	**534**	**786**	**23**	**82**
								proportion (%)	10.41	0.37	65.99	0.00	0.00	1.09	8.29	12.21	0.37	1.27
C. tibialis	Baiwang Mt.	Aug-14	*R. pseudoacacia*	17 (10)	267	28	206	33	0	7	0	0	7	0	0	18	0	1
		May-Jul-15	*R. pseudoacacia*	54 (20)	841	442	208	191	0	0	0	0	130	9	0	52	0	0
			total	**71 (30)**	**1108**	**470**	**414**	**224**	**0**	**7**	**0**	**0**	**137**	**9**	**0**	**70**	**0**	**1**
								proportion (%)	0	3.13	0	0	61.16	4.02	0	31.25	0	0.44
D. baccarum	Langfang	Jul-12	*Boehmeria siamensis, R. pseudoacacia*	18 (5)	502	298	84	120	108	0	0	0	0	0	2	0	10	0
		Jul-15	*Z. mays*	13 (10)	291	161	38	92	60	0	0	32	0	0	0	0	0	0
			total	**31 (15)**	**793**	**459**	**122**	**212**	**168**	**0**	**0**	**32**	**0**	**0**	**2**	**0**	**10**	**0**

								proportion (%)	79.25	0	0	15.09	0	0	0.94	0	4.72	0
A. chinensis	Langfang	Jul-12	*Zizyphus jujuba*	2 (1)	30	24	0	6	0	0	0	0	0	0	0	6	0	0
								proportion (%)	0	0	0	0	0	0	0	100	0	0
