# Supplementary material for: Seasonal parasitism and host specificity of Trissolcus japonicus in northern China
Source: J Pest Sci (2004). 2017 Apr 18;90(4):1127–41. doi: 10.1007/s10340-017-0863-y (PMC5544787; doi:10.1007/s10340-017-0863-y)
Supplement: Supplementary file 2 — Supplementary Table 2: Species composition of parasitoids reared from sentinel egg masses of Halymorpha halys and three non-target species exposed at various sites in 2014 (PDF 35 kb) [file 10340_2017_863_MOESM2_ESM.pdf]

										Parasitoid species composition							
**Non-target species**	**Location**	**Exposure month**	**# egg masses exposed /recollected**	**# eggs taken into rearing**	**# em. nymphs**	**# dead eggs**	**# parasitoids emerged**	**% total parasitism**	***Trissolcus japonicus***	***Trissolcus cultratus***	***Trissolcus plautiae***	***Trissolcus tumidus***	***Telenomus* sp.**	***Anastatus* sp.**	***Ooencyrtus* sp.**	***Acroclisoides* sp.**
Menida violacea	Lengquan village	June	49/47	668	152	144	372	55.7	278	0	75	6	12	1	0	0
		July	36/35	498	278	119	101	20.3	76	0	0	0	7	18	0	0
	Beianhe village	July	11/11	144	44	48	52	36.1	45	0	0	0	6	1	0	0
	Yangtai Mountain	June/July	10/10	138	75	37	26	18.8	14	0	0	0	0	12	0	0
	Sujiatuo village	June/July	11/10	141	74	55	12	8.5	6	0	0	0	0	0	6	0
	Fragrant Hills	June-Aug.	12/12	167	137	17	13	7.8	13	0	0	0	0	0	0	0
	Baiwang Mountain	May-Aug.	15/13	178	98	63	11	6.2	7	4	0	0	0	0	0	0
		∑	144/138	1,934	858	483	587	30.4	439	4	75	6	25	32	6	0
								%	74.8	0.7	12.8	1	4.3	5.5	1	0
Dolycoris baccarum	Lengquan village	June/July	42/42	1,114	333	634	149	13.4	105	0	2	0	24	15	3	0
	Beianhe village	June/July	8/8	179	10	85	84	46.9	42	0	0	12	20	10	0	0
	Yangtai Mountain	June/July	2/2	42	41	1	0	0	0	0	0	0	0	0	0	0
	Sujiatuo village	June/July	4/4	115	63	52	0	0	0	0	0	0	0	0	0	0
	Fragrant Hills	June/July	7/6	168	67	101	0	0	0	0	0	0	0	0	0	0
	Baiwang Mountain	Jun/July/Sep.	9/8	140	69	71	0	0	0	0	0	0	0	0	0	0
		∑	72/70	1,758	583	944	233	13.3	147	0	2	12	44	25	3	0
								%	**63.1**	**0**	**0.9**	**5.2**	**18.9**	**10.7**	**1.2**	**0**
Carbula eoa	Lengquan village	Aug.	32/32	430	219	100	111	25.8	42	42	0	0	14	0	13	0
	Beianhe village	Sep.	5/5	64	51	13	0	0	0	0	0	0	0	0	0	0
	Yangtai Mountain	Aug.	6/6	80	59	21	0	0	0	0	0	0	0	0	0	0
	Baiwang Mountain	Sep.	6/6	84	14	19	51	60.7	0	0	0	0	0	5	46	0
		∑	49/49	658	343	153	162	24.6	42	42	0	0	14	5	59	0
								%	**25.9**	**25.9**	**0**	**0**	**8.6**	**3.1**	**36.4**	**0**
Halyomorpha halys	Lengquan village	March	6/5	140	58	82	0	0	0	0	0	0	0	0	0	0
		April	28/23	620	475	145	0	0	0	0	0	0	0	0	0	0
		May	31/24	320	171	77	72	22.5	72	0	0	0	0	0	0	0
		June	30/28	354	77	147	130	36.7	130	0	0	0	0	0	0	0
		July	80/78	647	170	203	274	42.4	274	0	0	0	0	0	0	0
		Aug	37/37	433	49	126	258	59.6	233	20	0	0	0	5	0	0

	Sep.	1/1	28	9	19	0	0	0	0	0	0	0	0	0	0
Beianhe village	May	15/14	195	154	41	0	0	0	0	0	0	0	0	0	0
	June	16/16	200	0	32	168	84	158	9	0	0	1	0	0	0
	July	6/5	51	0	1	50	98	50	0	0	0	0	0	0	0
	Aug	2/2	26	0	1	25	96.2	0	25	0	0	0	0	0	0
Yantai Mountain	May	14/13	162	34	120	8	4.9	2	0	6	0	0	0	0	0
	June	8/6	108	50	31	27	25	27	0	0	0	0	0	0	0
	July	8/7	82	0	56	26	31.7	0	0	0	0	0	26	0	0
	Aug	3/3	17	14	3	0	0	0	0	0	0	0	0	0	0
Sujiatuo village	May	14/13	183	28	55	100	54.6	78	20	0	0	0	0	2	0
	June	16/15	190	88	77	25	13.2	25	0	0	0	0	0	0	0
	July	8/8	105	27	37	41	39.1	8	0	33	0	0	0	0	0
Fragrant Hills	May	8/5	56	28	28	0	0	0	0	0	0	0	0	0	0
	June	13/13	165	93	72	0	0	0	0	0	0	0	0	0	0
	July	12/12	154	45	67	42	27.3	37	5	0	0	0	0	0	0
	August	9/9	91	0	13	78	85.7	78	0	0	0	0	0	0	0
Baiwang Mountain	May	14/10	56	25	3	0	0	0	0	0	0	0	0	0	0
	June	21/21	187	38	16	133	71.1	132	0	0	0	0	1	0	0
	July	20/19	265	0	42	223	84.2	209	0	0	0	0	10	4	0
	∑	420/387	4835	1633	1494	1680	**34.7**	1513	79	39	0	1	42	6	0
							%	**90.06**	**4.70**	**2.32**	**0.00**	**0.06**	**2.50**	**0.36**	**0.00**
